# Outcome and Toxicity of Moderately Hypofractionated Post-Prostatectomy Radiotherapy: A Retrospective Study

**DOI:** 10.3390/medsci13040315

**Published:** 2025-12-12

**Authors:** Rocchina Vilella, Fiorella D’Auria, Luciana Valvano, Barbara D’Andrea, Antonietta Montagna, Giovanni Castaldo, Ilaria Benevento, Angela Pia Solazzo, Manuela Botte, Grazia Lazzari, Teodora Statuto, Luciana Rago

**Affiliations:** 1 Laboratory of Clinical Research and Advanced Diagnostics, Centro di Riferimento Oncologico della Basilicata (IRCCS-CROB), 85028 Rionero in Vulture, PZ, Italy; rocchina.vilella@crob.it (R.V.); luciana.valvano@crob.it (L.V.); teodora.statuto@crob.it (T.S.); 2 Laboratory of Clinical Pathology, Centro di Riferimento Oncologico della Basilicata (IRCCS-CROB), 85028 Rionero in Vulture, PZ, Italy; 3 Radiotherapy Unit, Centro di Riferimento Oncologico della Basilicata (IRCCS-CROB), 85028 Rionero in Vulture, PZ, Italy; barbara.dandrea@crob.it (B.D.); antonietta.montagna@crob.it (A.M.); giovanni.castaldo@crob.it (G.C.); ilaria.benevento@crob.it (I.B.); angela.solazzo@crob.it (A.P.S.); grazia.lazzari@crob.it (G.L.); luciana.rago@crob.it (L.R.); 4 Radiology Unit, Centro di Riferimento Oncologico della Basilicata (IRCCS-CROB), 85028 Rionero in Vulture, PZ, Italy; manuela.botte@crob.it

**Keywords:** moderate hypofractionation, post-prostatectomy, prostate cancer, radiotherapy, survival outcomes, toxicity, whole pelvis

## Abstract

**Background**: In this study, we retrospectively analyzed clinical and toxicity outcomes of 67 prostate cancer (PCa) patients undergoing moderately hypofractionated radiotherapy (RT) after prostatectomy, with adjuvant or salvage intent. **Methods**: Irradiation was delivered by volumetric modulated arc therapy. The median follow-up was 48 months. The 3- and 5-year biochemical relapse-free survival rates were 80% and 69%. The RT schedule consisted of a median total dose of 67.5 Gy with a median number of 25 fractions and a median fraction dose of 2.7 Gy to the prostate bed (PB) and 60% of patients simultaneously received whole pelvis irradiation (WP; fraction dose: 1.8 Gy, median total dose of 46.8 Gy). **Results**: The rate of acute toxicity was 54% for gastrointestinal (GI) and 36% for genitourinary (GU). No grade 3 acute toxicity was observed. Late toxicity was as follows: G1, G2, and G3 GI events in 25.5%, 3.6%, and 1.8% of the cases, respectively; G1, G2, and G3 GU events in 37.1%, 11.1%, and 7.4%, respectively. The toxicity-free survival (TFS) curves showed a different trend for acute and late toxicity. TFS was significantly associated with RT volume, except for acute GI toxicity. Specifically, the concomitant irradiation of PB and WP appeared to be a significant risk factor for late GI and GU toxicity (*p* = 0.029 and *p* = 0.012, respectively). **Conclusions**: At the 48-month median timepoint considered by our study, postoperative hypofractionated RT achieved promising results in terms of clinical outcomes with acceptable toxicity. Only the irradiated volume seems to be an important predictor for toxicity.

## 1. Introduction

Hypofractionation is a well-established radiotherapy (RT) treatment modality in the intact prostate cancer (PCa) setting, whose effectiveness and tolerability have also been extensively studied in comparison to conventional RT [1,2,3,4,5]. It is an RT option able to reduce costs and time for both hospital facilities and patients [6]. The picture relating to hypofractionated RT after radical prostatectomy (RP) is less delineated and clear. Post-prostatectomy, RT can be administered as an adjuvant to prevent recurrence in patients with adverse disease features [7], or as a salvage therapeutic option for patients who experience biochemical relapse [8].

In the post-prostatectomy setting, two prospective randomized phase 3 studies have compared conventional (1.8–2 Gy) and hypofractionated RT (2.5–2.625 Gy) [9,10]. Specifically, these studies showed that there were no significant differences in terms of genitourinary (GU) and gastrointestinal (GI) toxic effects between these two fractionation schedules.

Some retrospective studies comparing these two types of fractionation in PCa patients after RP [3,11,12,13,14,15,16,17] reported that hypofractionated RT leads to promising biochemical control and toxicity comparable to conventional RT. In contrast, Cozzarini et al. found a higher risk of severe late urinary toxicity in the group of patients treated with hypofractionated RT compared with conventional fractionation [18].

Recent phase 1/2 studies on hypofractionated RT in the postoperative setting highlighted acceptable rates of acute and late GU and GI toxicity, as well as good clinical efficacy, despite using heterogeneous dose schemes and toxicity scoring systems [19,20,21,22,23].

In this context, our retrospective single-center study aimed to analyze the clinical outcomes and the GU and GI toxicity profile of patients exposed to moderate post-prostatectomy hypofractionated RT, with adjuvant or salvage intent. We also assessed the possible influence of the irradiated volumes and patient comorbidities.

## 2. Materials and Methods

### 2.1. Patients’ Characteristics

Our retrospective single-institution study was approved by the local ethics committee (Comitato Etico Unico Regionale per la Basilicata, approval No. 20240048815 of 5 December 2024) and was conducted following the principles of the Declaration of Helsinki. The patients included in the study underwent moderately hypofractionated RT from 2016 to 2021 with adjuvant or salvage intent. Additional inclusion criteria for patient selection were as follows:

- PCa treated with RP with or without lymph node dissection;
- pT2-pT4 disease with or without positive surgical margins;
- No distant metastases;
- RT technique: volumetric modulated arc therapy (VMAT);
- Concomitant whole pelvis (WP) irradiation allowed;
- Androgen-deprivation therapy (ADT) allowed.

The physician, based on the patients’ risk factors, decided whether to subject them to adjuvant therapy after prostatectomy. This information was taken from the medical records of each patient, from which we retrospectively drew the clinical information. In particular, patients received adjuvant RT (ART) within 6 months of prostatectomy if they had at least one of the following risk factors: pT ≥ 3, N1, or R1. Salvage RT (SRT) was administered in the presence of a prostate-specific antigen (PSA) ≥ 0.2 ng/mL on at least 2 consecutive measurements after surgery. Only one SRT patient had a PSA value ≤ 0.2 ng/mL; 22 patients had a PSA value ≤ 0.5 ng/mL, while the remaining 11 had PSA values ≥ 0.5 ng/mL. Based on the patient’s risk factors, including a higher Gleason score, lymph node involvement, positive surgical margins, and early biochemical recurrence, the irradiated volumes included either the prostate bed (PB) only or the PB concomitantly with the WP (PB + WP) in order to prophylactically irradiate all pelvic lymph nodes. When the pelvis was irradiated, a hypofractionated simultaneous integrated boost (SIB) was delivered to the PB.

The GU and GI RT toxicity was recorded according to the Common Terminology Criteria for Adverse Events (CTCAE) version 5.0. For acute toxicity, we considered events occurring within 120 days from the start of RT treatment.

### 2.2. Radiotherapy Planning Parameters

The clinical target volume 1 (CTV1) consisted of the PB and CTV2 consisted of the WP, including all pelvic lymph nodes. The planning target volume 1 (PTV1) was obtained by adding 5 mm in all directions and 3 mm posteriorly to CTV1. The PTV2 was obtained by adding 5 mm in all directions to CTV2.

The RT schedule consisted of a median total dose to PTV1 of 67.5 Gy (range, 62–70) delivered in a median number of 25 fractions (range, 20–28) with a median daily dose of 2.7 Gy (range, 2.5–3.1). The median total dose to PTV2 was 46.8 Gy, (range 45–50.4) with a daily dose of 1.8 Gy.

Organs at risk (OAR) dose constraints were as follows: for the bladder V48 Gy < 50%, V65 Gy < 25%; for the rectum V52 Gy < 35%, V65 Gy < 20%; for the femoral heads V43 Gy < 10%; for the bowel V39 Gy < 195 cc; for penile bulb Dmean < 43 Gy.

### 2.3. Statistical Analysis

Descriptive statistics were performed on categorical and continuous variables. Data were reported as numbers and percentages for categorical variables and as a median and range for continuous variables. The Kolmogorov–Smirnov test was performed to check the normal distribution for continuous variables. The frequency distribution of the onset of acute and late GI and GU toxicities was analyzed using the chi-square (χ^2^) test. The Wilcoxon signed-rank test was used to evaluate, for each patient, the differences between the acute and late GI/GU toxicity grades. As appropriate, the Mann–Whitney U test and χ^2^ test were used to compare pathological characteristics between the ART and SRT groups.

The endpoints of the study were as follows: overall survival (OS), biochemical relapse-free survival (BRFS), disease metastasis-free survival (DMFS), and toxicity-free survival (TFS). OS was defined as the time from the start of RT treatment to death, for any cause, or last follow-up; BRFS indicated biochemical failure and was defined as the interval from the last day of RT to the PSA increment (≥0.2 ng/mL), or death, or last follow-up; DMFS was defined as the interval from the last day of RT to the onset of metastasis, or death, or last follow-up; TFS was defined as the time between the first day of RT and the date of GI and GU toxicity occurrence.

Survival curves, stratified for the different clinical variables (ART vs. SRT, ADT during RT, irradiated volumes, hypertension, and diabetes) were generated by the Kaplan–Meier method and the differences between groups were evaluated by the long-rank test. The Cox regression model was used to assess significant prognostic factors for OS, BRFS, DMFS, and TFS. Specifically, the following parameters were evaluated: age; PSA level before RT; T stage; Gleason score; surgical margin; adjuvant or salvage therapy; RT volumes (PB vs. PB + WP); ADT during RT; hypertension (HTN); and diabetes. Only the relevant results will be reported.

A *p*-value ≤ 0.05 was considered statistically significant. All statistical analyses were performed using SPSS software (IBM Corp., New York, NY, USA), version 28. GraphPad Prism software (version 10.3.1) was used to generate all of the graphs.

## 3. Results

Between January 2016 and December 2021, 67 surgically resected PCa patients treated with hypofractionated RT were considered. A summary of the patients’ characteristics is shown in Table 1. The median interval between surgery and the start of RT was 8 months overall (range, 2–228 months), 48 months in the SRT group (range, 4–228 months), and 4 months in the ART group (range, 4–18 months).

In Table 2, we report the base-line pathological characteristics related to the ART and SRT groups. 

### 3.1. Clinical Outcomes

**Figure 1:**
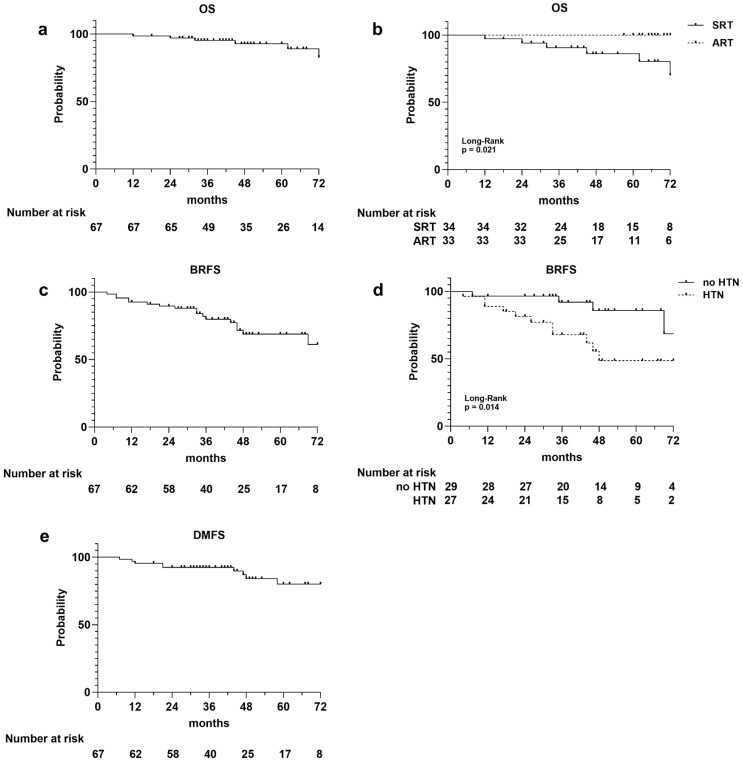
Kaplan–Meier survival curves illustrating the following: overall survival (OS) curves for entire population (**a**), for adjuvant (ART) and salvage (SRT) radiotherapy cohorts (**b**); biochemical relapse-free survival (BRFS) for the entire population (**c**), and for patients with and without hypertension (**d**); disease metastasis-free survival (DMFS) for entire population (**e**). Number at risk is reported at the bottom of each curve.

A total of six deaths occurred, three of which were not PCa related, resulting in a 1-, 3-, and 5-year OS rate of 99%, 95%, and 93%, respectively (Figure 1a). The survival curves stratified for ART versus SRT were statistically different (*p* = 0.021), and all the registered deaths occurred in the SRT group (Figure 1b). There was no difference in the OS rate between HTN and non-HTN patients (*p* = 0.631).

Considering biochemical relapse, it occurred in 17 (25%) patients, 8 in the ART group and 9 in the SRT group, with a 1-, 3-, and 5-year BRFS rates of 93%, 80%, and 69%, respectively (Figure 1c). The median PSA value at biochemical recurrence was 12.5 ng/mL. There was no difference in the BRFS rate between ART and SRT (*p* = 0.888). Conversely, the BRFS curve’s stratification for HTN showed an increased risk of biochemical relapse in patients with hypertension (*p* = 0.014; Figure 1d), which was confirmed by univariate analysis (Cox regression model, HR 3.78; IC% 1.20–11.96; *p* = 0.023). The 3- and 5-year BRFS rates were 88% and 83% for the non-HTN group and 68% and 49% for patients with HTN.

Metastatic recurrence occurred in 10 patients (two lung, four bone, and four lymph node metastases), with 1-, 2-, and 3-year DMFS rates of 96%, 93%, and 80%, respectively (Figure 1e). The DMFS curves stratified for the adjuvant and salvage cohorts of patients did not show differences between the two groups, while a slight difference was observed between the HTN and non-HTN groups, it did not reach statistical significance (*p* = 0.133). A significant association with DMFS was observed for the serum PSA level measured before RT (univariate Cox regression model, HR 1.40; IC% 1.12–1.75; *p* = 0.003).

The OS, BRFS, and DMFS rates were not influenced by ADT during RT, irradiated RT volume, and diabetes.

### 3.2. Toxicity Evaluation

The number of patients that experienced RT, acute and late GI and GU toxicities, their relative grades, and the main symptoms observed are summarized in Table 3.

Acute GI and GU toxicities of any grade were observed in 54% and 36% of cases, respectively. None of the patients experienced grade 3 acute toxicity. Concerning GI toxicity, the rate of late toxicity was significantly lower (*p* = 0.005, Supplementary Figure S1a), and a significant decrease in toxicity grades from the acute to the late phase was observed (*p* = 0.001, Supplementary Figure S1b). Notably, after symptom-related therapies, over 50% of patients with acute GI toxicity showed complete symptom resolution, and 14 patients experienced an improvement in toxicity grades from 2 to 1. Only one patient developed severe toxicity (grade 3) 14 months after PB irradiation, specifically, an extensive rectal ulcer that required a blood transfusion and martial therapy. 

Conversely, the incidence rate of acute GU toxicity was significantly lower (*p* = 0.027, Supplementary Figure S1a). Late GU toxicity was detected in 56% of patients (new-onset or worsened cases after RT). In particular, we observed four cases of grade 3 late GU toxicity. Three patients developed gross hematuria: one occurred 27 months after PB irradiation, and the other two occurred 13 and 11 months after PB + WP irradiation. Additionally, one patient experienced bladder lithiasis and urinary tract obstruction 28 months after PB + WP irradiation, which required cystolitholapaxy after urethrotomy. This approach proved to be definitive.

The baseline urinary function (pre-RT) is reported in Table 4.

**Figure 2:**
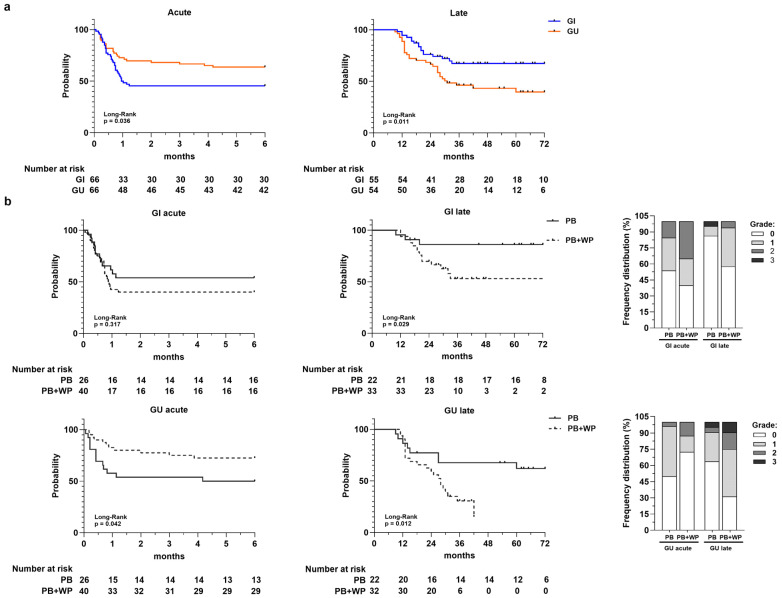
Panel (**a**) Toxicity-free survival curves for acute GI and GU toxicity and for late GI and GU toxicity. Number at risk is reported at the bottom of each curve. Panel (**b**) Toxicity-free survival curves for acute and late GI and GU toxicities according to radiotherapy volumes: prostate bed (PB) vs. prostate bed + whole pelvis (PB + WP). Number at risk is reported at the bottom of each curve. In the stacked bar charts are the frequency distributions of acute and late GI/GU toxicity grades in PB and PB + WP groups, respectively.

The Kaplan–Meier TFS curves (Figure 2a) showed a different trend for acute and late toxicity, highlighting an increase in cumulative probability to develop acute GI toxicity early compared to GU toxicity (*p* = 0.036; median GI = 1 month; median GU = not reached), unlike late toxicity. The overall late TFS rate at 1-, 3-, and 5-years was 95%, 68%, and 68% for GI and 89%, 46%, and 40% for GU, respectively. Patients with late GU toxicity had significantly lower TFS compared to those that developed late GI toxicity, which tended to reach a plateau around 3 years (*p* = 0.011; median TFS GU = 31 months; median TFS GI = not reached).

When TFS was stratified according to irradiated volumes (Figure 2b), we observed a statistically significant lower acute GU TFS in patients that received PB-only irradiation compared to those underwent concomitant PB and WP irradiation (*p* = 0.042). Conversely, for GI and GU late toxicity, we observed a significant difference between the two groups (PB vs. PB + WP) with a much lower TFS in the PB + WP group. The frequency distributions of acute and late toxicities along with their relative grades for the PB and PB + WP groups are shown in the stacked bar charts of Figure 2b.

A similar trend was observed when the TFS curves were stratified for ART and SRT in the PB and PB + WP groups (Supplementary Figure S2), while no difference was found in patients divided for ART and SRT only (Supplementary Figure S3).

In the univariate analysis (Cox model), the only variable significantly associated with acute and late TFS was the irradiated volumes (Supplementary Table S1), except for acute GI toxicity. Specifically, the concomitant irradiation of PB and WP significantly reduced the risk of developing acute GU toxicity (HR = 0.45; 95% IC = 0.20–1.00; *p* = 0.049); this is in contrast to what occurs for late GI and GU toxicity, where it appeared to be a statistically significant risk factor (late GI HR = 3.67, 95% IC = 1.05–12.88, *p* = 0.042; late GU HR = 2.87, 95% IC = 1.20–6.87, *p* = 0.018).

## 4. Discussion

At 5 years, 93% of patients were alive, and three of the six deaths observed were unrelated to PCa. The 5-year BRFS rate was 69%. These data are in line with the outcome trend of other studies that, like ours, include both patients treated with moderate hypofractionation in salvage and adjuvant regimens [12,24]. We found that patients with HTN have a higher risk of biochemical relapse, which translates into lower 3- and 5-year BRFS rates. The association between preexisting hypertension and higher rates of BCR after radical prostatectomy has been reported in several studies [25,26,27,28]. Ohwaki et al. demonstrated that BCR was significantly associated with a higher systolic blood pressure and marginally associated with higher diastolic blood pressure [26]. The use of antihypertensive medication was found to be inversely associated with the incidence of prostate cancer and was also linked to improved PCa-specific survival [29]. Alashkhsm et al. showed that inhibitors of the renin–angiotensin system (angiotensin-converting enzyme inhibitors) and angiotensin receptor blockers may be associated with lower rates of BCR after radical radiotherapy combined with adjuvant/neoadjuvant hormone treatment [30]. Future research is needed in this area to explore the potential associations between blood pressure elevation, the use of antihypertensive medications, and PCa prognosis more fully.

In our cohort, we observed a significant association between increasing pre-RT PSA values and worse DMFS. Similar findings have been reported in recent studies, in particular, for pre-RT PSA values greater than 0.5 ng/mL [31,32].

In terms of toxicity, in the acute phase, we recorded a greater number (27%) of grade 2 GI (diarrhea and anal pain) compared to GU (9%: cystitis, urinary frequency, and tract pain), but no grade ≥3. These data reflect what has been reported by Moll et al. [16] in their hypofractionation group of VMAT-treated patients, which, like our patients, included a proportion of patients (60%) who also underwent pelvic lymph node irradiation.

As regards late toxicity, we detected a reversal of trend, with more cases of GU toxicity than GI. In fact, more than 50% of acute GI cases resolved over time. During the follow-up period, we recorded six cases of late GU grade 2 toxicity, one already present in the acute phase and five new or worsened (median onset: 14 months), and four cases of grade 3 (median onset: 20 months). Consequently, while the 5-year TFS is 68% for GI, it is 40% for GU. For the detection of these late cases of severe late urinary toxicity, a long patient follow-up is required [17,33].

Surprisingly, while for GI and GU late toxicity we documented a worsening of TFS curves in patients concomitantly irradiated on the WP and the PB, in the acute phase, the GU-TFS curve was better than in patients who received the PB-only irradiation. This is possibly because of the increased attention to urinary toxicity over time; we recorded more pre-RT GU toxicities for the WP + PB group of patients, many of whom were the most recently recruited (years 2020–2021). Consequently, many acute GU toxicities, above all, urinary incontinence, which were already present before RT, were registered as grade 0 (as no RT-related toxicity). The baseline urinary function differences between the two groups of patients may have introduced bias that led to the acute GU-TFS curve being lower in PB + WP compared to PB only. For that reason, the retrospective nature of the study is one of our limitations. As regards the frequency, grade 2 cases of acute GU toxicity almost tripled in the WP + PB group compared to the PB group.

When considering studies comparing treatment that only exposed the prostate/prostate bed to radiation with those that also exposed the WP, we find different data in terms of toxicity. As regards the curative-intent setting (patients with prostate cancer), in the study of Murthy et al., pelvic irradiation using hypofractionated IMRT resulted in increased grade 2 or higher late GU toxicity as compared to prostate-only RT [34]. Instead, as reported by Parry et al., the inclusion of pelvic lymph nodes in radiation fields for high-risk or locally advanced PCa is not associated with increased GI or GU toxicity at the 3-year follow-up [35].

In the postoperative setting, early GI side effects ≥G2 were significantly more numerous in local + pelvis RT compared to only local RT, as well as for late GU morbidity [36]. Conversely, significant differences between the WP RT + ADT group and ADT group (with equal irradiation on the PB) were recorded for grade ≥2 acute GI toxicities but not for GU toxicities. Furthermore, there were no significant differences between the groups in late grade ≥2 GU or GI adverse events [37].

We acknowledge that our study presents some limitations. First, the retrospective nature of the study introduced information bias related to incomplete medical records, including missing data on the ADT duration, comorbidities, antihypertensive medications, and baseline urinary alterations in early enrolled patients. Second, differences in the duration of follow-up between early vs. late-treated patients affected the number of recorded severe late GU toxicity events, as longer follow-up is required for these toxicities to emerge. Additionally, the small size of the study cohort and the lack of a conventional fractionation control group limited the statistical power of our analyses. Therefore, further investigations with larger patient populations are needed to confirm and validate our findings.

The scarcity of robust evidence in the literature on GU and GI toxicity following post-prostatectomy hypofractionated RT underscores the value of our single-center study, which offers real-world data that can meaningfully inform and guide future prospective research.

In conclusion, our study highlights the effectiveness of moderately hypofractionated treatment (median daily dose of 2.7 Gy administered in 25 median number of fractions) in the postoperative settings in terms of OS, BRFS, and DMFS, despite its retrospective nature’s limitation. We also recorded a few serious adverse events. For the detection of any grade 2/3 adverse events, attention must be paid to long follow-up and to irradiation of the pelvis, which may be a risk factor for shorter TFS in the late GU and GI setting.

## Figures and Tables

**Table 1:** Patients’ characteristics (*n* = 67).

Median age, years (range)	70 (51–88)
Median follow-up, months (range)	48 (12–96)
Pathological characteristics	
T stage, N (%)	
pT2	22 (32.8)
pT3a	14 (20.9)
pT3b	28 (41.8)
pT4	1 (1.5)
Unknown	2 (3)
N stage, N (%)	
N0	39 (58)
N1	7 (10.5)
NX	14 (21)
Unknown	7 (10.5)
Surgical margin, N (%)	
R0	10 (14)
R1	38 (57)
Unknown	19 (28)
Gleason score, N (%)	
5	1 (1.5)
6	15 (22.3)
7	31 (46.3)
8	13 (19.4)
9	5 (7.5)
Unknown	2 (3)
Median PSA before RT, ng/mL (range)	0.40 (0.01–12.45)
Treatment characteristics	
ART, N (%)	33 (49)
SRT, N (%)	34 (51)
ADT * during RT, N (%)	23 (34)
Clinical target volume, N (%)	
PB	27 (40)
PB + WP	40 (60)
Comorbidities	
Hypertension, N (%)	27 (40)
Diabetes, N (%)	10 (15)

PSA: prostate-specific antigen; RT = radiotherapy; ART = adjuvant radiotherapy; SRT = salvage radiotherapy; ADT = androgen-deprivation therapy; PB = prostate bed; PB + WP = prostate bed + whole pelvis. * Antiandrogens (*n* = 7, 4 in the SRT group and 3 in the ART group, respectively); luteinizing hormone-releasing hormone (LHRH) agonist/antagonist (*n* = 10, 5 in the SRT group and 5 in the ART group, respectively); antiandrogens + LHRH agonist/antagonist (*n* = 13, 7 in the SRT group and 6 in the ART group, respectively); data not available in 3 patients. The criteria of administration were the presence of BRFS and/or a higher Gleason score and/or high-risk pathological features.

**Table 2:** Comparison between the pathological data of the ART and SRT groups.

Pathological Characteristics	SRT	ART	*p*-Value
Median PSA before RT, ng/mL (range)	0.675 (0.17–7.50)	0.090 (0.01–12.4)	<0.001 *
Gleason score, N (%)			0.255
5 (2 + 3)	1 (2.9)	0 (0.0)	
6 (3 + 3)	11 (32.4)	4 (12.9)	
7 (3 + 4)	9 (26.5)	6 (19.4)	
7 (4 + 3)	8 (23.5)	8 (25.8)	
8 (5 + 3)	0 (0.0)	2 (6.5)	
8 (4 + 4)	4 (11.8)	6 (19.4)	
9 (4 + 5)	1 (2.9)	3 (9.7)	
9 (5 + 4)	0 (0.0)	1 (3.2)	
T stage, N (%)			0.030 ^#^
pT2	16 (47.1)	6 (18.2)	
PT3a	4 (11.8)	10 (30.3)	
pT3b	12 (35.3)	16 (48.5)	
pT4	0 (0.0)	1 (3.0)	
Unknown	2 (5.9)	0 (0.0)	
N stage, N (%)			0.258
N0	16 (47.1)	22 (66.7)	
N1	3 (8.8)	4 (12.1)	
NX	10 (29.4)	5 (15.2)	
Unknown	5 (14.7)	2 (6.1)	
Surgical margin, N (%)			<0.001 ^#^
R0	9 (26.5)	1 (3.0)	
R1	9 (26.5)	29 (87.9)	
Unknown	16 (47.1)	3 (9.1)	

* Mann–Whitney U test; ^#^ χ^2^ test.

**Table 3:** Acute and late GI and GU RT toxicities according to the CTCAE (v. 5.0).

Toxicity		Grade 0	Grade 1	Grade 2	Grade 3	Total
Acute GI	N (%)	30 (45.4)	18 (27.3)	18 (27.3)	0	66
	Symptoms		anal pain (*n* = 2); acute diarrhea (*n* = 10); constipation (*n* = 1); rectal tenesmus (*n* = 5).	proctitis and diarrhea (*n* = 1); rectal tenesmus and anal pain (*n* = 4); rectal tenesmus and diarrhea (*n* = 5); rectal tenesmus and bloating (*n* = 1); severe anal pain (*n* = 1); severe constipation (*n* = 1); severe diarrhea (*n* = 4); severe rectal tenesmus (*n* = 1).		
Acute GU	N (%)	42 (63.6)	18 (27.3)	6 (9.1)	0	66
	Symptoms		cystitis (*n* = 5); cystitis and urinary incontinence (*n* = 1); cystitis and weak urine flow (*n* = 1); dysuria (*n* = 2); nocturia (*n* = 1); pollakiuria (*n* = 2); pollakiuria and nocturia (*n* = 2); urinary incontinence (*n* = 3); weak urine flow (*n* = 1).	cystitis requiring medication (*n* = 2); cystitis + dysuria + pollakiuria + balanoposthitis (*n* = 1); dysuria and strangury (*n* = 1); pollakiuria requiring medication and nocturia (*n* = 1); pollakiuria and dysuria (*n* = 1).		
Late GI	N (%)	38 (69.1)	14 (25.5)	2(3.6)	1 (1.8)	55
	Symptoms		constipated bowel (*n* = 1); diarrhea (*n* = 2); mucorrhea (*n* = 1); proctitis (*n* = 2); proctitis and rectal bleeding (*n* = 2); rectal tenesmus (*n* = 2); rectal tenesmus + diarrhea + mucorrhea (*n* = 1); rectal tenesmus + constipated bowel + rectal bleeding (*n* = 1); telangiectasias (*n* = 1); undefined (*n* = 1).	frequent proctitis (*n* = 1); frequent proctitis and rectal bleeding (*n* = 1).	rectal bleeding and rectal ulcer (*n* = 1).	
Late GU	N (%)	24 (44.4)	20 (37.1)	6 (11.1)	4 (7.4)	54
	Symptoms		dysuria (*n* = 1); dysuria and urinary incontinence (*n* = 1); hematuria (*n* = 3); hemorrhagic cystitis (*n* = 1); nocturia and urinary urgency (*n* = 1); pollakiuria (*n* = 1); pollakiuria and urinary urgency (*n* = 1); pubic hyperesthesia and strangury (*n* = 1); urinary incontinence (*n* = 7); weak urine flow (*n* = 1); weak urine flow and urinary incontinence (*n* = 1); undefined (*n* = 1).	cystitis requiring medication (*n* = 1); hematuria (*n* = 1); hemorrhagic cystitis and urinary incontinence (*n* = 2); severe urinary incontinence (*n* = 2).	bladder lithiasis and urinary tract obstruction (*n* = 1); gross hematuria (*n* = 3).	

Grade 0 = no toxicity; GI: gastrointestinal; GU: genitourinary.

**Table 4:** Baseline (pre-RT) urinary function.

Adverse Event ^#^	PB Group (*n* = 27)	PB + WP Group (*n* = 38 *)	Total (*n* = 65)
Urinary incontinence, N (%)	11 (40.7)	30 (78.9)	41 (63.1)
Urinary tract pain, N (%)	2 (7.4)	1 (2.6)	3 (4.6)
Urinary urgency, N (%)	3 (11.1)	5 (13.2)	8 (12.3)

^#^ All adverse events reported prior to the start of RT were grade 1. * We do not have any pre-RT urine function data for two patients in the PB + WP group.

## Data Availability

The original contributions presented in this study are included in the article/Supplementary Material. Further inquiries can be directed to the corresponding author.

## Supplementary Materials

The following supporting information can be downloaded at: https://www.mdpi.com/article/10.3390/medsci13040315/s1. Figure S1: Frequency distribution of late and acute toxicity. Figure S2: Toxicity-free survival curves estimate for acute and late GI and GU toxicities stratified for adjuvant and salvage radiotherapy in prostate bed (PB) and prostate bed + whole pelvis (PB + WP) groups. Figure S3: Toxicity-free survival curves estimate for acute and late GI and GU toxicities stratified for adjuvant and salvage cohorts. Table S1: Baseline (pre-RT) urinary function. Table S2: Univariate analysis for acute and late GI and GU toxicities.
