# Supplementary figures and images for: Outcome and Toxicity of Moderately Hypofractionated Post-Prostatectomy Radiotherapy: A Retrospective Study
Source: Med Sci (Basel). 2025 Dec 12;13(4):315. doi: 10.3390/medsci13040315 (PMC12735228; doi:10.3390/medsci13040315)

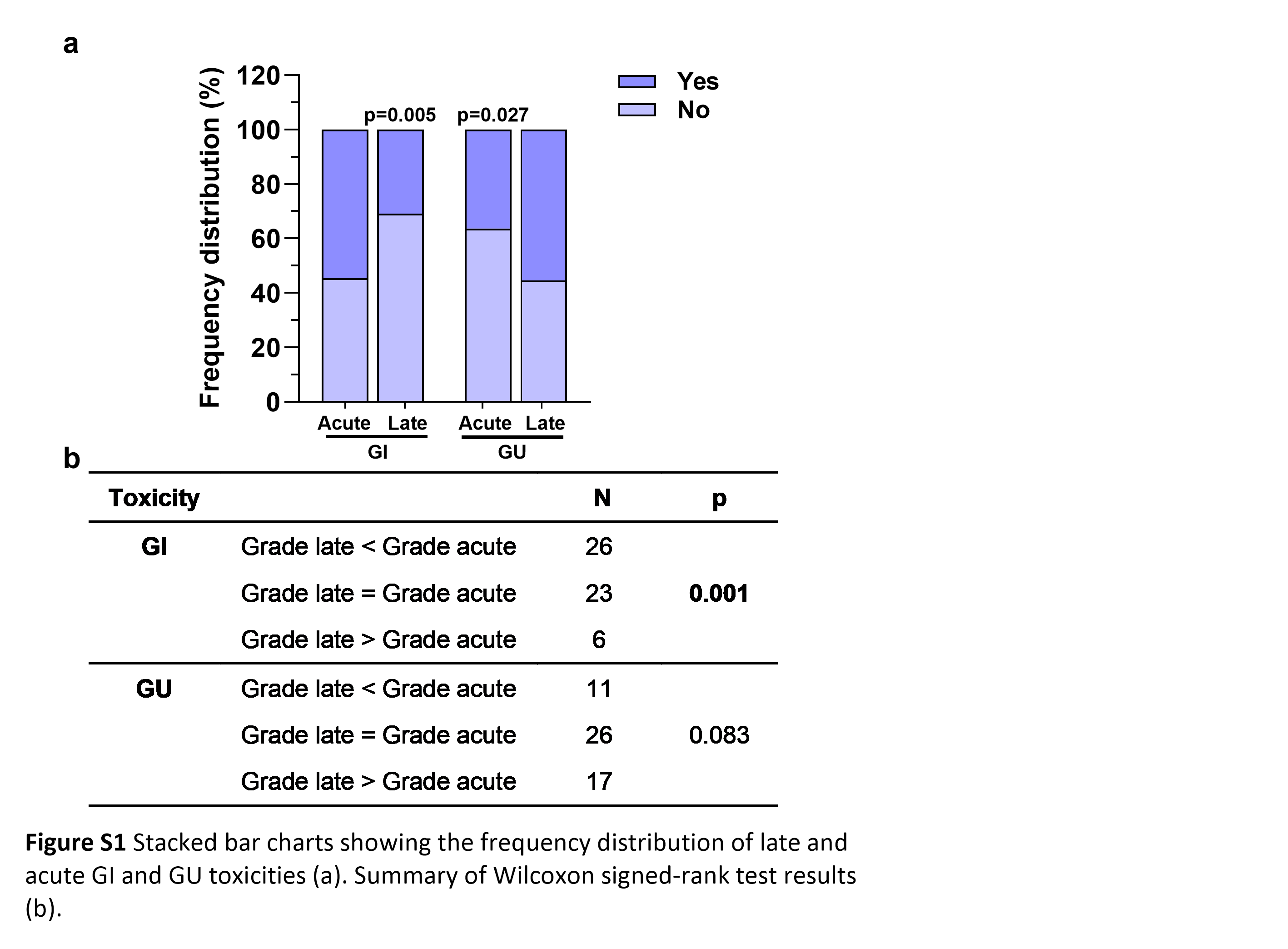

Supplement: Supplementary file 1 [file medsci-13-00315-s001.zip › Figure S1.TIF]

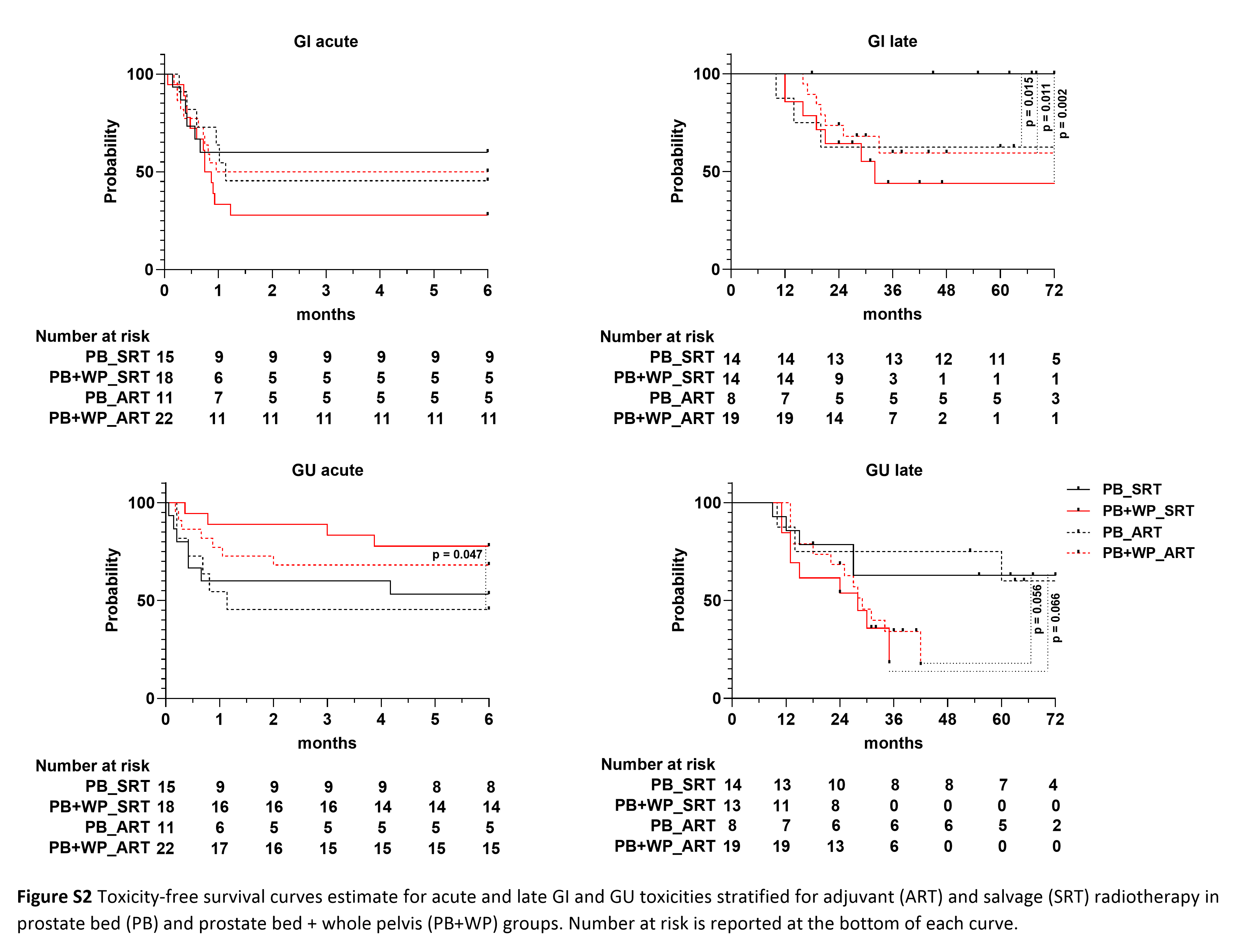

Supplement: Supplementary file 1 [file medsci-13-00315-s001.zip › Figure S2.TIF]

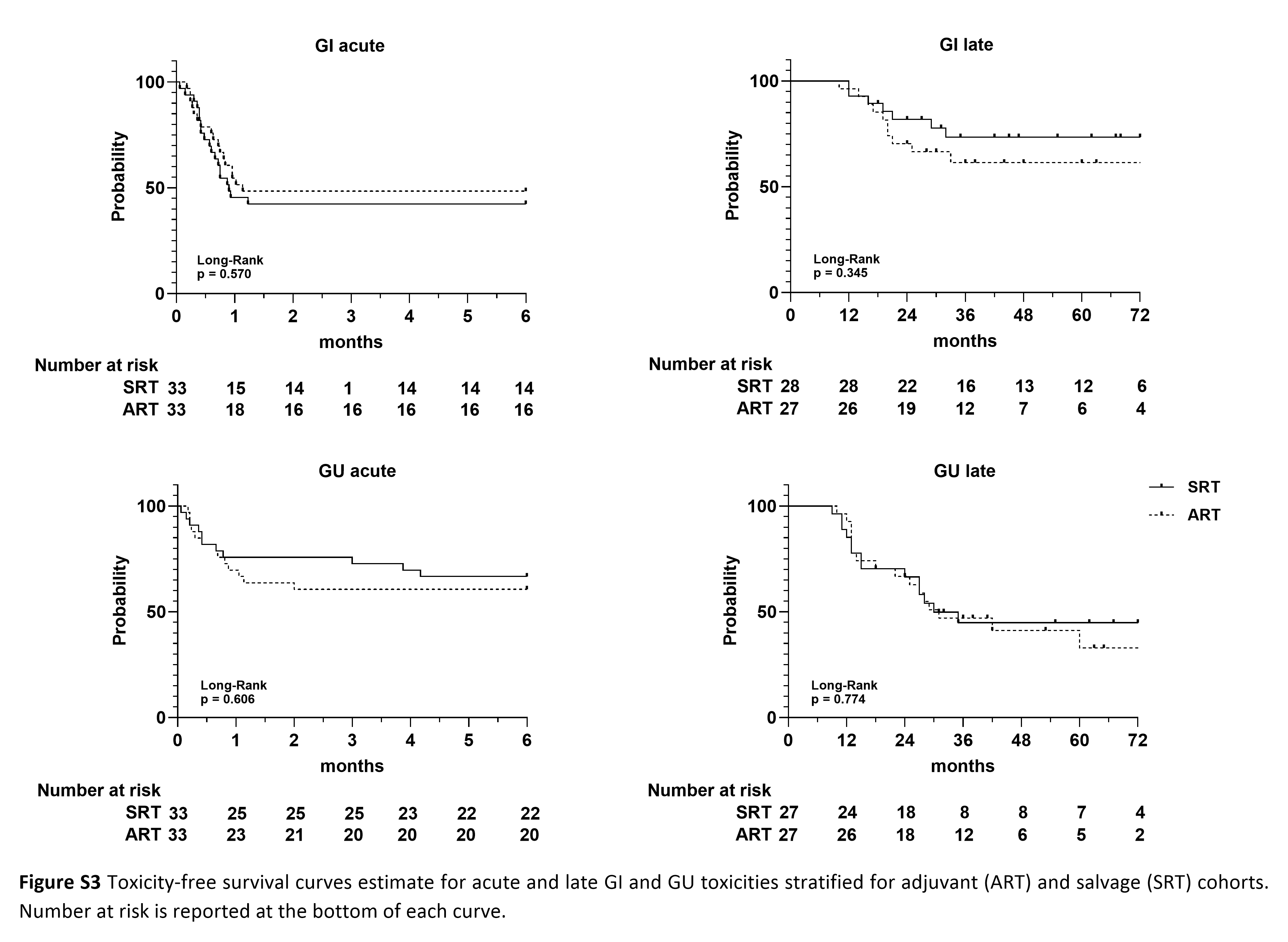

Supplement: Supplementary file 1 [file medsci-13-00315-s001.zip › Figure S3.TIF]

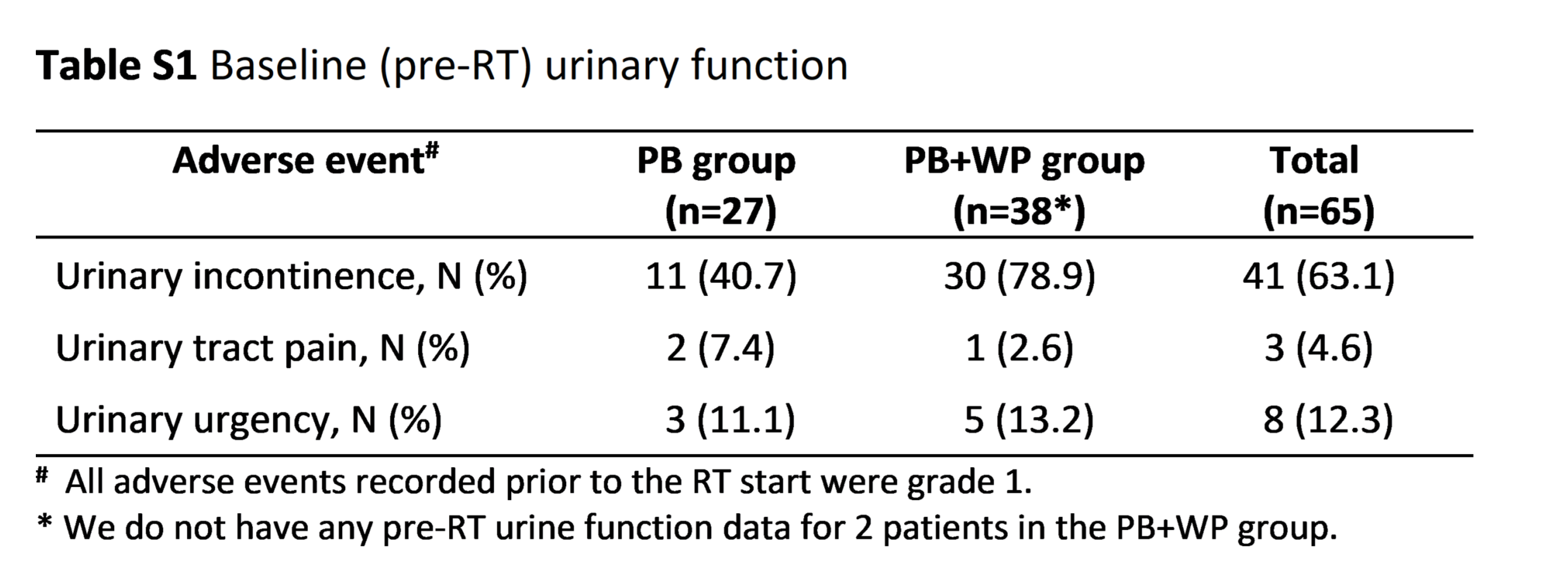

Supplement: Supplementary file 1 [file medsci-13-00315-s001.zip › Table S1.TIF]

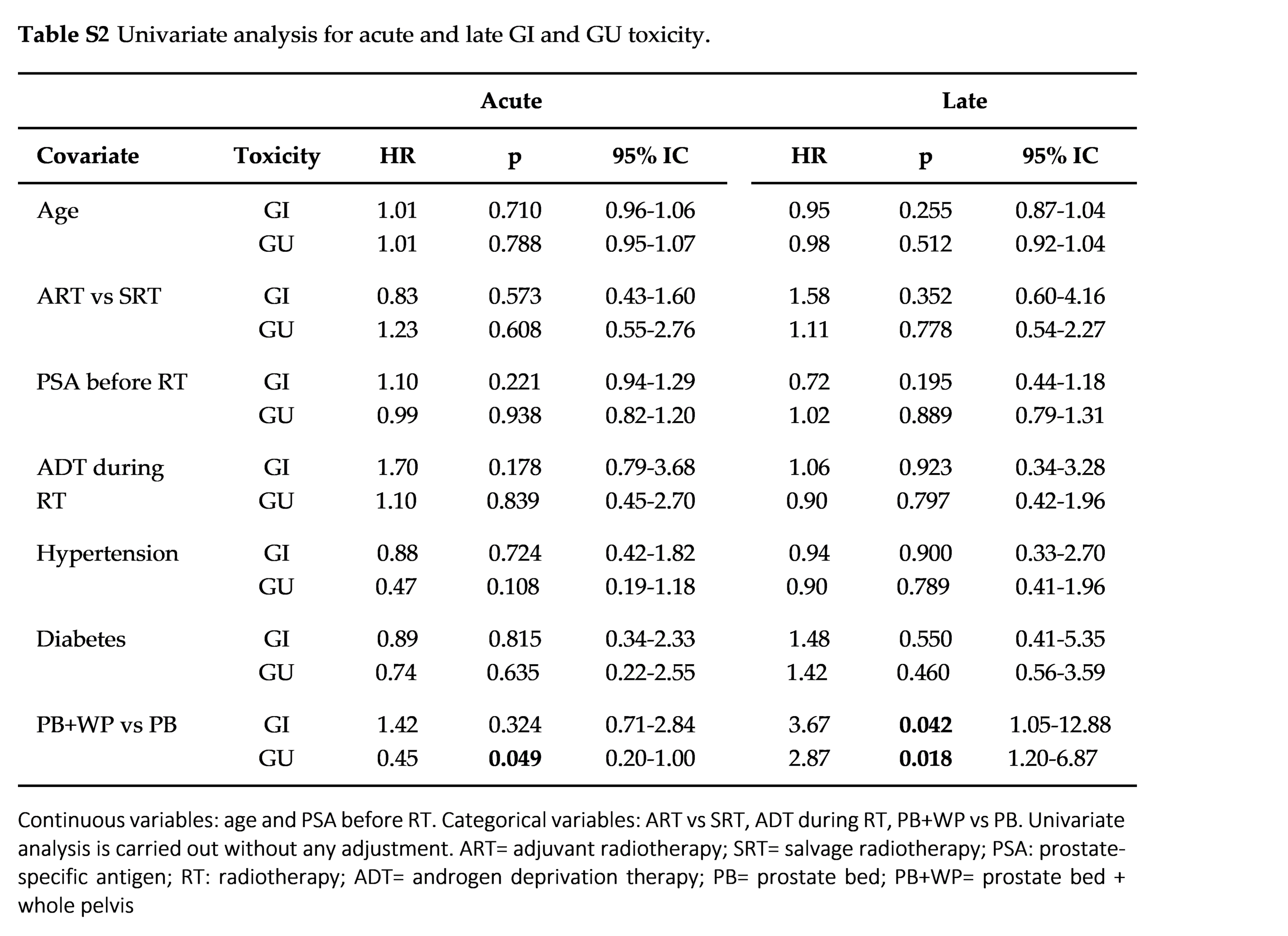

Supplement: Supplementary file 1 [file medsci-13-00315-s001.zip › Table S2.tif]
